# Impact of Smoking and Sociodemographic Factors on Self-Reported Chronic Obstructive Pulmonary Disease Among U.S. Adults: A Retrospective Study

**DOI:** 10.7759/cureus.89053

**Published:** 2025-07-30

**Authors:** Shrivatsa Bhat, Maryam Shehhi, Maira Jalil, Sukriti Azad, Joy D Doshi, Sameena Safarullah

**Affiliations:** 1 Internal Medicine, Namma Clinic Lakshminagara, Udupi, IND; 2 Internal Medicine, Khalifa University, Abu Dhabi, ARE; 3 Internal Medicine, University of Debrecen, Faculty of Medicine, Debrecen, HUN; 4 Internal Medicine, Government of National Capital Territory of Delhi, Rohini, IND; 5 Emergency Medicine, University Hospital Lewisham, Lewisham and Greenwich NHS Trust, London, GBR; 6 Internal Medicine, Al-Azhar Medical College and Super Specialty Hospital, Kerala, IND

**Keywords:** behavioral risk factor surveillance system (brfss), chronic obstructive pulmonary disease (copd), odds ratio, retrospective study, smoking

## Abstract

Introduction: Chronic obstructive pulmonary disease (COPD), including chronic bronchitis and emphysema, is a major health concern in the U.S., with smoking as its leading risk factor. Understanding the role of demographic and socioeconomic factors is crucial for targeted public health strategies.

Aim: This study investigated the link between smoking and self-reported COPD among U.S. adults, also assessing the impact of demographic variables (age, gender, and race) and socioeconomic variables (education and income) on this association.

Methods: A retrospective study was conducted using data from the 2022 Behavioral Risk Factor Surveillance System (BRFSS). The outcome variable was self-reported COPD status, and the main exposure was smoking status (Yes/No). Control variables included age, gender, race, education level, and annual income. Statistical analyses included cross-tabulations, and odds ratios (ORs) with 95% confidence intervals (CIs) were calculated to evaluate associations.

Results: Smokers had significantly higher odds of reporting COPD compared to non-smokers (OR: 3.879; 95% CI: 3.7809-3.9792). The strongest associations were seen in individuals aged 45-64 years (OR: 5.241) and females (OR: 4.337). White non-Hispanic smokers had the highest odds among racial groups (OR: 4.118). Smokers with lower education levels (OR: 3.059) and incomes below $50,000 (OR: 3.103) also showed elevated odds of reporting COPD.

Conclusion: Smoking is strongly associated with self-reported COPD across demographic and socioeconomic groups. The findings highlight increased risk among middle-aged adults, females, White non-Hispanic individuals, and those with lower income and education. Public health efforts should prioritize cessation programs tailored to these high-risk populations.

## Introduction

Chronic obstructive pulmonary disease (COPD), encompassing emphysema and chronic bronchitis, is a leading cause of long-term morbidity and mortality globally. It is also set to be the third highest cause of death in the world, with more than three million deaths a year due to the disease, mainly occurring in low- and middle-income countries [1]. Cigarette smoking is the single most important and avoidable cause of COPD and accounts for as much as 90% of cases in developed nations [2]. Smoking tobacco leads to chronic inflammation of the airways and irreversible limitation of airflow, causing irreversible respiratory disability and decreased quality of life [3].

In addition to individual health, smoking and smoking-related illness such as COPD are a heavy burden on public health systems. Smokers cause over eight million deaths worldwide every year, and the economic impact is over $1.4 trillion per annum in healthcare and lost productivity [4]. COPD-related healthcare alone in the United States is over $49 billion per annum, with smoking cessation programs ranked as extremely cost-effective interventions [5].

Although smoking is the major modifiable risk factor, demographic and socioeconomic determinants have a strong impact on disease prevalence, progression, and outcomes. Lower educational levels, lower income, and reduced access to healthcare services disproportionately affect these populations [6]. Racial and ethnic disparities further contribute to these trends, frequently resulting from variations in exposure, diagnosis, and access to treatment.

The Behavioral Risk Factor Surveillance System (BRFSS), a nationwide representative survey, facilitates detailed exploration of these correlations. This article makes use of the 2022 BRFSS dataset to examine the connection between current smoking status and reported diagnosis of COPD, emphysema, or chronic bronchitis. The paper also investigates the way demographic and socioeconomic factors influence this relationship with the intent of informing public health interventions aimed at both behavior and structural risk factors.

## Materials and methods

A retrospective original research study was conducted using the BRFSS database [7]. Data for this study were extracted on April 1, 2025 and analyzed. Since the BRFSS database contains de-identified public data, this research qualifies as non-human participant research. As a result, ethics committee approval was not required.

Data were extracted using the BRFSS WEB-Enabled Analysis tool (WEAT) for the year 2022. The disease variable in this study was COPD, emphysema or chronic bronchitis disease (COPD, emphysema, or chronic bronchitis (CHCCOPD3)), while the risk factor variable was smoking status (calculated variable for adults who are current smokers (_RFSMOK3)). Control variables included demographic and socioeconomic characteristics. Demographic characteristics were Age (_AGE_G) categorized into four groups (18-24, 25-44, 45-64, 65+); gender (SEX), categorized as male and female; and race (_RACEGR), categorized as White (non-Hispanic), Black (non-Hispanic), and Hispanic/other races. Socioeconomic characteristics included educational level (EDUCA), categorized as basic education (high school or less) and advanced education (some college or higher), and Annual income (_INCOMG1), categorized as <$50,000 and >$50,000. Participants who responded to the selected disease, risk factor, and control variables were included in the study, ensuring completeness of data for analysis.

Descriptive statistics, including numbers and percentages, were generated for each variable using cross-tabulation in the BRFSS Web-Enabled Analysis Tool. The data were exported to Microsoft Excel (Microsoft Corporation, USA) for organization and further processing. Statistical analyses were performed using R version 4.3.1 (R Core Team, Vienna, Austria). The results were expressed as odds ratio (OR) with 95% confidence intervals (CIs). A p-value of <0.05 was considered statistically significant.

## Results

A total of 407,848 individuals have participated in the 2022 BRFSS survey and were part of our study. In this study, no records were excluded to ensure an inclusive dataset. This comprehensive dataset strengthens the validity of our analysis by encompassing a broader population across numerous demographics and regions in the United States.

Table 1 represents the overall odds of reporting COPD, emphysema, or chronic bronchitis among current smokers versus non-smoking participants. Among current smokers, 20.8% disclosed having one of these conditions, compared to 6.4% among non-smokers. The calculated OR was 3.879 (95% CI: 3.78-3.98), demonstrating that current smokers were relatively four times more likely to report chronic respiratory disease than non-smokers. This statistically distinctive association underscores the high correlation between chronic respiratory conditions and smoking.

**Table 1:** Prevalence of current smokers in the participants with self-reported COPD (chronic obstructive pulmonary disease), emphysema, or chronic bronchitis disease *Significant CI = confidence interval, OR = odd’s ratio

Current smoker	Yes	COPD, emphysema, or chronic bronchitis disease		OR (CI)
		Yes	No	3.879* (3.7809, 3.9792)
		10,329 (20.8%)	39,221 (79.2%)	
	No	22,780 (6.4%)	33,5518 (93.6%)	

The respondents' demographic characteristics are shown in Table 2. The respondents were classified based on age group, gender, and race/ethnicity. The largest proportion of the respondents fell under the 65+ age group, followed by the 45-64 years age category, which exhibited the highest risk with an OR of 5.241, indicating an over fivefold increase in likelihood of respiratory disease among smokers in this category. Similarly, female respondents had an OR of 4.337, reflecting greater vulnerability compared to male respondents (OR = 3.475). Age and gender appear to alter the strength of the correlation between smoking and disease. In terms of race and ethnicity, White, non-Hispanic smokers demonstrated higher odds (OR = 4.118) compared to Black non-Hispanic (OR = 3.072) and Hispanic/other groups (OR = 3.515). These results suggest that racial variations in disease risk that may reflect differences in exposure, diagnosis, or access to healthcare.

**Table 2:** Prevalence of current smokers in the participants with self-reported COPD (chronic obstructive pulmonary disease), emphysema, or chronic bronchitis disease based on the demographic characteristics *Significant CI = confidence interval, OR = odd’s ratio

Demographic characteristics	Current smoker	N	Participants with COPD, emphysema, or chronic bronchitis disease	Participants without COPD, emphysema, or chronic bronchitis disease	OR (CI)
Age groups					
18-24 years	Yes	1,602	75 (4.7%)	1,527 (95.3%)	3.524* (2.6885,4.5717)
	No	23,211	319 (1.4%)	22,892 (98.6%)	
25-44 years	Yes	14,566	1,132 (7.8%)	13,434 (92.2%)	4.158* (3.8429,4.4965)
	No	83,413	1,657 (2%)	81,756 (98%)	
45-64 years	Yes	21,242	4,918 (23.2%)	16,324 (76.8%)	5.241* (5.0294,5.4599)
	No	114,839	6,243 (5.4%)	108,596 (94.6%)	
65+ years	Yes	12,140	4,204 (34.6%)	7,936 (65.4%)	4.448* (4.2674,4.6366)
	No	136,835	14,561 (10.6%)	122,274 (89.4%)	
Gender					
Male	Yes	25,090	4,343 (17.3%)	20,747 (82.7%)	3.475* (3.3418,3.6125)
	No	167,481	9,516 (5.7%)	157,965 (94.3%)	
Female	Yes	24,460	5,986 (24.5%)	18,474 (75.5%)	4.337* (4.1913,4.4883)
	No	190,817	13,264 (7%)	177,553 (93%)	
Race					
White, non-Hispanic	Yes	35,026	8,119 (23.2%)	26,907 (76.8%)	4.118* (3.9989,4.2394)
	No	261,906	17,883 (6.8%)	244,023 (93.2%)	
Black, non-Hispanic	Yes	4,382	747 (17%)	3,635 (83%)	3.072* (2.7953,3.3734)
	No	26,920	1,688 (6.3%)	25,232 (93.7%)	
Hispanic / Others	Yes	8,600	1,145 (13.3%)	7,455 (86.7%)	3.515* (3.2609,3.7866)
	No	59,423	2,488 (4.2%)	56,935 (95.8%)	

Table 3 outlines the relationship between the socioeconomic factors and the prevalence of the disease. Smokers with basic educational levels had an OR of 3.059, whereas those with advanced education maintained elevated odds (OR = 4.028), which may reflect enhanced access to diagnosis and health literacy among participants with higher education. Furthermore, income exhibited a gradient effect; smokers with <$50,000 earnings had an OR of 3.103 versus 3.362 for participants above this threshold. Interestingly, participants who smoke, even those with higher incomes, demonstrated a greater increased disease risk, emphasizing that smoking remains a fundamental contributor regardless of socioeconomic advantages.

**Table 3:** Prevalence of current smokers in the participants with self-reported COPD (chronic obstructive pulmonary disease), emphysema, or chronic bronchitis disease based on the socioeconomic characteristics *Significant CI = confidence interval, OR = odd’s ratio

Socioeconomic characteristics	Current smoker	N	Participants with COPD, emphysema, or chronic bronchitis disease	Participants without COPD, emphysema,or chronic bronchitis disease	OR (CI)
Education level					
Basic education	Yes	23,652	5,533 (23.4%)	18,119 (76.6%)	3.059* (2.9465,3.1748)
	No	98,055	8,901 (9.1%)	89,154 (90.9%)	
Advanced education	Yes	25,740	4,757 (18.5%)	20,983 (81.5%)	4.028* (3.8852,4.1749)
	No	258,830	13,792 (5.3%)	245,038 (94.7%)	
Annual income					
Less than $50,000	Yes	25,751	6,702 (26%)	19,049 (74%)	3.103* (2.9989,3.2099)
	No	111,926	11,399 (10.2%)	100,527 (89.8%)	
Greater than $50,000	Yes	16,315	1,919 (11.8%)	14,396 (88.2%)	3.362* (3.1854,3.5469)
	No	179,687	6,853 (3.8%)	172,834 (96.2%)	

In summary, the OR analysis consistently demonstrates that smoking is a robust and independent predictor of self-reported COPD, emphysema, or chronic bronchitis among all subgroups. Increased ORs among middle-aged smokers, women, in addition to low- and high-income earners, underscore that the relationship between smoking and disease crosses demographic and economic boundaries.

## Discussion

A retrospective study conducted on April 1, 2025, using the 2022 BRFSS dataset, a critical tool for public health surveillance, investigated the link between current smoking and self-reported diagnoses of COPD, emphysema, or chronic bronchitis in the United States. The analysis confirmed a strong association, with current smokers having significantly higher odds of developing these conditions than non-smokers. Prevalence was notably higher among females and increased with age, peaking in the 65+ group, although the odds were most elevated in the 45-64 age groups. Socioeconomic factors revealed a higher prevalence in individuals with basic education and incomes below $50,000, yet higher odds among those with advanced education and incomes above $50,000, suggesting complex risk interactions.

Cigarette smoking is a primary cause of COPD, emphysema, and chronic bronchitis, driving parallel epidemics that pose a major global health challenge, particularly in regions with high tobacco use [8]. It induces COPD through emphysema, involving lung parenchyma destruction and chronic bronchitis, marked by airway narrowing due to chronic inflammation and fibrosis [9]. Cigarette smoke’s reactive oxygen species, along with components such as tar, cause oxidative stress and promote lung inflammation and damage [10]. These components trigger pathways that lead to mucus hypersecretion, airway remodeling, and alveolar destruction [11]. Increased goblet and inflammatory cells, such as leukocytes and macrophages, in the airways cause obstruction [12]. Smoking-induced lung damage mimics aging with self-sustaining structural changes [13]. A dose-response relationship links higher smoking intensity to greater COPD severity [14].

Table 1 of the 2022 BRFSS data underscores the strong association between current smoking and COPD prevalence, highlighting the pivotal role of smoking in chronic respiratory disease. This critical link was evidenced by a pattern corroborated by large-scale studies. Specifically, current smokers exhibited a 15.2% age-adjusted prevalence compared to only 2.8% in never-smokers, indicating a fivefold increased likelihood of reporting these conditions [15]. Reinforcing this, a National Health and Nutrition Examination Survey (NHANES) analysis (1999-2018) reported a 12.6% COPD prevalence among smokers versus 4.1% in never-smokers, further confirming the elevated risk associated with smoking [16]. Similarly, PATH Study analyses showed that current cigarette users face a threefold higher COPD risk (RR = 3.00, 95% CI: 2.37-3.80) even after adjusting for demographics, other tobacco products, or dual use with e-cigarettes, solidifying the robust connection between smoking and COPD [17]. Furthermore, a BRFSS study (2016-2017) confirmed that current cigarette smokers have a significantly higher risk of chronic lung diseases than never smokers [18].

Table 2 of the BRFSS data revealed elevated COPD prevalence among current smokers, particularly those aged 45-64 years, females, and White non-Hispanics, reflecting demographic trends in disease burden [19]. Notably, females exhibited greater susceptibility, likely influenced by smoking patterns or genetic predispositions, which aligns with projections of increasing COPD prevalence in women [20]. This trend is further supported by the higher prevalence among White non-Hispanics, consistent with studies reporting elevated COPD rates in this group [21]. While global data often show a higher prevalence among men, North America’s age-related increases, possibly driven by regional smoking behaviors, highlight unique regional patterns [22].

Table 3 reveals that current smokers with lower education and income had a significantly higher COPD prevalence than those with higher socioeconomic status. This elevated risk is often linked to limited education, which reduces health literacy and sustains smoking habits [23]. These educational disparities contribute to broader socioeconomic challenges, increasing exposure to environmental irritants, and perpetuating smoking behaviors [24]. In rural areas, poverty further amplifies COPD prevalence by compounding these socioeconomic disadvantages [25]. Similarly, low income restricts access to healthcare, heightens exposure to respiratory irritants, and delays diagnosis and treatment [26]. These interconnected factors highlight the urgent need for targeted interventions such as smoking cessation programs and improved healthcare access to reduce COPD disparities among lower socioeconomic groups.

This study had certain limitations. As a telephone-based survey, individuals without landline access or those unavailable during contact may have introduced a selection bias. Reliance on self-reported data, rather than clinical evaluations, may affect diagnostic accuracy. In addition, the BRFSS dataset lacks details on the type, severity, and timing of COPD diagnosis, which could limit the depth of analysis. Finally, the retrospective observational design allowed only the identification of associations, not causal relationships; prospective studies are needed to establish causality between smoking and chronic respiratory diseases.

## Conclusions

This study confirmed a strong association between current smoking status and increased COPD prevalence across diverse demographic and socioeconomic groups. The risk was notably higher among females, those aged 45-64 years, White non-Hispanics, and individuals with lower education and incomes, underscoring the complex interplay of smoking with social determinants. These findings highlight the urgent need for targeted interventions to address disparities in the COPD burden, particularly in vulnerable populations. Future research should prioritize prospective studies to elucidate the biological and social mechanisms driving these associations, including the roles of smoking intensity, environmental exposure, and healthcare access. Such investigations are crucial for developing evidence-based smoking cessation programs and preventive strategies tailored to demographic and socioeconomic profiles. By addressing these factors, public health efforts can reduce COPD prevalence and mitigate its disproportionate impact on lower socioeconomic groups.
